# Letter to the editor: The first tick-borne encephalitis case in the Netherlands: reflections and a note of caution

**DOI:** 10.2807/1560-7917.ES.2016.21.39.30355

**Published:** 2016-09-29

**Authors:** Jan Clement, Katrien Lagrou, Veroniek Saegeman, Piet Maes, Marc van Ranst

**Affiliations:** 1 University Hospital Leuven, Laboratory of Clinical Virology, Rega Institute for Medical Research, Leuven, Belgium; 2 University Hospital Leuven, National Reference Centre for Lyme Borreliosis, Leuven, Belgium; 3 University Hospital Leuven, Department of Laboratory Medicine, Leuven, Belgium

**Keywords:** Tick-borne encephalitis, TBE, neutralization tests, flavivirus infections cerebrospinal fluid, CSF testing, the Netherlands

**To the editor: **We wish to offer some cautionary remarks concerning the report by de Graaf et al. [1] about the first human tick-borne encephalitis (TBE) infection in the Netherlands, acquired in June 2016 (and not in July, as incorrectly mentioned in the title). At first sight, this case, apparently proven by ELISA and confirmed by neutralisation tests (NT), seems extremely convincing, especially as it occurred after a bite from a local tick (species not mentioned) that was later found by qRT-PCR to be infected with a recently discovered Dutch TBE virus (TBEV).

However, this case could only be called 100% waterproof, if (i) the *Eurosurveillance* reader was given verifiable taxonomic data about the novel Salland TBEV and its relationship with other pathogenic TBEV and (ii) a convincing degree of homology was demonstrated between the TBEV isolated from the tick and the patient. Neither of these conditions was fulfilled in this Rapid communication. Admittedly, condition (ii), although an unquestionable paradigm for zoonotic infections, will be hard to fulfil in any forthcoming TBE case because TBEV is nearly always cleared from blood and cerebrospinal fluid (CSF) already at the start of the second TBE phase, i.e. before the patient is admitted with neurologic complications [2,3]. Consequently, and against our own expectations, RT-PCR has not become the ultimate tool for physicians attending a putative TBE case [4], as was again demonstrated in this case.

Thus, physicians have to rely solely on serological techniques, which have a number of flaws: TBEV shares common antigenic sites in its E protein with several other pathogenic flaviviruses, resulting in false-positive results in IgG and even IgM ELISA [2,4-7]. The ELISA seropositivity in the presented case could thus in theory be ascribed to the patient’s yellow fever vaccination 11 years earlier [2,6,7]. Moreover, the specificity of gold standard NT, considered hitherto as sacrosanct, has recently also been called into question: in an animal study, four of five louping ill–infected sheep and two of 17 sera from West Nile virus (WNV)-infected horses, collected in TBE non-endemic regions and tested at British and German reference laboratories, reacted positive in TBE ELISA and even in TBE NT [7].

There are however still other, and more obvious, question marks concerning the evidence of true TBEV infection in the case under discussion. Firstly, two titres obtained in the NT remained unchanged (1/640). Although the crucial interval in days is not exactly specified (on days 24 and 36?), serological immobility is surprising for acute TBE when neutralising antibodies can be expected to increase. It is however compatible with a status of post-vaccination cross-protection. Secondly, and most importantly, CSF was only IgG-positive, while IgM was lacking. IgM-positivity in CSF is, however, paramount for diagnosing TBE and other flaviviral infections such as West Nile fever, to the extent that CSF IgG is not even considered, nor required for diagnosis, certainly not in a patient with prior contact with flaviviruses [2]. Moreover, IgM-positivity in CSF is almost invariably present by the 10th day of TBE illness [2] and peaking between day 9 and week 6 [3].

Finally, CSF findings are supportive of TBE diagnosis, when there is (i) pleocytosis with predominance of segmented granulocytes over lymphocytes, (ii) impairment of the blood–CSF barrier (increased CSF/serum albumin ratio), and (iii) intrathecal synthesis of immunoglobulins, predominantly of IgM [2]. None of these techniques were applied in the current case. As for the pleocytosis, an almost exclusive mononuclear cell reaction was found, which, to our knowledge, is highly unusual for TBE.

Since CSF, for unclear reasons, remained only IgG-positive, determining the CSF/serum IgG ratio could be helpful in proving or disproving the TBE origin of this diagnostically challenging case.
